# Attention-Deficit/Hyperactivity Disorder (ADHD): Integrating the MOXO-dCPT with an Eye Tracker Enhances Diagnostic Precision

**DOI:** 10.3390/s20216386

**Published:** 2020-11-09

**Authors:** Tomer Elbaum, Yoram Braw, Astar Lev, Yuri Rassovsky

**Affiliations:** 1 Department of Industrial Engineering and Management, Ariel University, Ariel 40700, Israel; tomere@ariel.ac.il; 2 Department of Psychology, Ariel University, Ariel 40700, Israel; 3 Department of Psychology, Bar-Ilan University, Ramat Gan 5290002, Israel; astarastarlev@gmail.com (A.L.); or yurir@ucla.edu (Y.R.); 4 Leslie and Susan Gonda (Goldschmied) Multidisciplinary Brain Research Center, Bar-Ilan University, Ramat Gan 5290002, Israel; 5 Department of Psychiatry and Biobehavioral Sciences, University of California, Los Angeles (UCLA), Los Angeles, CA 90095, USA

**Keywords:** attention-deficit/hyperactivity disorder (ADHD), continuous performance tests (CPT), MOXO-dCPT, eye movements

## Abstract

Clinical decision-making may be enhanced when combining psychophysiological sensors with computerized neuropsychological tests. The current study explored the utility of integrating an eye tracker with a commercially available continuous performance test (CPT), the MOXO-dCPT. As part of the study, the performance of adult attention-deficit/hyperactivity disorder (ADHD) patients and healthy controls (*n* = 43, *n* = 42, respectively) was compared in the integrated system. More specifically, the MOXO-dCPT has four stages, which differ in their combinations of ecological visual and auditory dynamic distractors. By exploring the participants’ performance in each of the stages, we were able to show that: (a) ADHD patients spend significantly more time gazing at irrelevant areas of interest (AOIs) compared to healthy controls; (b) visual distractors are particularly effective in impacting ADHD patients’ eye movements, suggesting their enhanced utility in diagnostic procedures; (c) combining gaze direction data and conventional CPT indices enhances group prediction, compared to the sole use of conventional indices. Overall, the findings indicate the utility of eye tracker-integrated CPTs and their enhanced diagnostic precision. They also suggest that the use of attention-grabbing visual distractors may be a promising path for the evolution of existing CPTs by shortening their duration and enhancing diagnostic precision.

## 1. Introduction

Attention-deficit/hyperactivity disorder (ADHD) is a neurodevelopmental disorder that is characterized by a “persistent pattern of inattention and/or hyperactivity-impulsivity that interferes with functioning or development” [1]. Though its prevalence decreases with age [2], adult ADHD is still a concern, as about 2.5% of adults are estimated to have this disorder [3]. This concern is fueled by the impact of ADHD on the emotional and cognitive functioning of patients [4,5] and, consequently, on their daily lives [6,7].

The current diagnostic procedure regarding ADHD is based on a clinical evaluation by a qualified clinician. As it is a multifaceted disorder, this usually entails collecting data from different sources, including clinical psychiatric interviews with both the individual and informant/s that may provide additional non-overlapping information (see review of 13 different national guidelines; [8]). Screening for comorbidity and a physical examination are also usually included, as well as rating scales, questionnaires, and cognitive testing. The latter is of value, as ADHD patients exhibit impairments in a variety of cognitive domains including, although not restricted to, attentional and executive functions [5]. Moreover, cognitive impairment is a major source of disability [9]. Establishing the cognitive profile of adult ADHD patients is therefore of clear importance and may aid both in diagnosing those suspected to have the disorder and in promoting individualized treatment of those that may benefit from it (e.g., neurocognitive remediation; [10]). Continuous performance tests (CPTs) are widely used cognitive tests that provide valuable information regarding key cognitive functions such as focused attention, impulsivity, and other relevant executive functions [11,12]. They include a rapid presentation of visual and/or auditory stimuli over a relatively prolonged time, during which examinees are requested to respond to certain stimuli while ignoring others (i.e., targets and non-targets). Thereby, CPTs simulate relevant environments that ADHD patients struggle with, such as academic studies [6]. These necessitate prolonged and passive reception of information with the occasional need to respond to external stimuli. They also mandate the participant to ignore both internal and environmental distractors.

Findings are mixed regarding the ability of CPTs to support clinical decision-making (see reviews; [11,13]). CPTs have good sensitivity but poor specificity for diagnosing ADHD [13], as exemplified by a recent study in which the Conners CPT3™ classified only 51.7% of adult ADHD patients as likely to have a disorder characterized by attention impairment [14]. However, there are studies which support the usefulness of CPTs to clarify the cognitive profile of examinees (e.g., [15]). Importantly, some have even found that CPTs enhanced diagnostic precision when combined with data derived from other sources. The integration of psychophysiological data with conventional CPT indices seems to be especially promising. For example, the quantified behavior test plus (QBT+), which assesses hyperactivity by tracking examinees’ head movement during the test, has shown initial promise [13]. This promise has been echoed in recent studies using measures such as functional magnetic resonance imaging, electroencephalography, and event-related potentials (fMRI, EEG, and ERPs, respectively; [16,17]). These efforts are part of a larger endeavor to establish biomarkers of psychiatric disorders, evident in an increase in research efforts and the establishment of criteria for using them in clinical practice [18,19].

Eye movement research has flourished since links between cognitive processes and eye movements were first described [20]. Eye movement analyses are currently used in studying various cognitive processes, as they provide unique information regarding these processes, and the equipment required is increasingly more affordable and convenient to use [21]. This is exemplified by studies of ADHD in the development of the anti-saccade task, a task in which a voluntary eye movement is made in the direction opposite to the side where a stimulus is presented. The difficulty that ADHD patients show in this task [22] parallels indications that deficits in inhibitory control (i.e., disinhibition) are a major element of the cognitive profile of ADHD [5,23]. This is particularly important when assessing adult patients, since they may have developed top-down control which masks their impairment while performing cognitive tests (e.g., avoiding responses to non-targets in CPTs). Eye movements are under lessened top-down control and may therefore be uniquely suited to unravel cognitive impairments that may otherwise escape detection. In other words, eye tracking is a particularly attractive biomarker which may provide unique information regarding ADHD-associated cognitive impairment [24]. Correspondingly, the potential of eye tracking has been increasingly investigated using experimentally built CPTs and the methodologically similar Go/No-go tasks (e.g., [25,26]). However, very few studies have integrated an eye tracker with a commercially available CPT. This may be related to the technological difficulty of integrating an eye tracker with an existing system, the need to approach commercial entities which may be hesitant about changes in their established CPT, financial considerations, or a combination of such factors. At any rate, this is regrettable as such studies could hasten the accumulation of knowledge and shorten the time for the adoption of this novel technology in daily clinical practice. This potential is exemplified in two studies. Fried, et al. [27] found that adult ADHD patients made more microsaccades (i.e., small, jerk-like eye movements during fixation) than controls in the test of variables of attention (TOVA), a widely used CPT. More recently, ADHD patients were found to spend significantly more time gazing at irrelevant field of view (FOV) regions compared to healthy controls in another CPT, the MOXO-dCPT [28]. This latter study utilized gaze location measures, indicating the allocation of visual attention to areas of interest (AOIs). As these measures can be collected with relative ease using the currently available eye trackers, they offer to clarify the cognitive profile of ADHD patients while hopefully enhancing diagnostic precision and illuminating a path for the evolution of existing CPTs.

The MOXO-dCPT has garnered significant research attention, mainly because it is based on an online platform and does not require the acquisition of special equipment (for recent studies, see [29,30,31,32,33,34,35,36,37]). Importantly, this CPT uses dynamic auditory and visual distractors, such as the barking of a dog, which imitates stimuli encountered in real life. These distractors aim to magnify the cognitive impairment of ADHD patients and therefore make their detection easier [38,39]. Eye movement analysis perfectly complements the MOXO-dCPT’s design, as it allows a straightforward determination of examinees’ distractibility. More specifically, gazing outside the location of CPT’s target/non-target stimuli (i.e., the task area at the center of the screen) indicates a less than optimal performance by the examinee. Therefore, highly relevant information can be obtained by capitalizing on the fact that participants’ gaze direction is an intuitive and easily obtainable eye movement measure.

As part of the current study, we assessed the utility of eye movement measures to differentiate ADHD patients and healthy controls and their potential to enhance the discrimination capacity of conventional MOXO-dCPT indices. Importantly, the differential utility of the CPT’s stages was assessed as part of the current study. These differ in the presence and type of distractors (i.e., no distractors, visual distractors, auditory distractors, and a combination of visual and auditory distractors). Thereby, they offer unique opportunities to enhance our theoretical understanding of ADHD and, consequently, to enhance the utility of CPTs. For example, while inattention seems to be partially independent of modality (i.e., evident in both visual- and auditory-based CPTs), response inhibition seems to be modality-specific [40]. Interestingly, initial evidence suggests that the MOXO-dCPT distractors differ in their ability to differentiate ADHD patients and healthy controls, with visual distractors and combined distractors (visual and auditory) increasing omission errors in the CPT [38,39]. However, studies are currently numbered, and we do not have data regarding the efficacy of the distractor type—as well the effect of combining visual and auditory distractors—on participants’ eye movements. This is unfortunate, since it may enable the construction of CPTs with enhanced diagnostic precision.

The current study explored these possibilities using a two-pronged approach. First, we divided the visual environment of the MOXO-dCPT into two regions (i.e., task AOI and distractibility AOI). We then assessed the allocation of attention by ADHD patients and healthy controls while they performed the MOXO-dCPT. We hypothesized that compared to healthy controls, ADHD patients would gaze for longer durations outside the screen area (i.e., distractibility AOI), which is irrelevant for success in the task. We also hypothesized that combining an eye movement measure with the conventional MOXO-dCPT indices would enhance the CPT’s diagnostic precision (i.e., diagnosis based on conventional indices). Second, we investigated the impact of distractor types (i.e., visual, auditory, or a combination of the two types of distractors) on ADHD patients’ performance in the CPT. This was done with a practical purpose in mind. More specifically, if similar or better utility will be found in the first stages of the CPT, later stages can be discarded without decreasing the CPT’s discrimination capacity (i.e., shortening the duration of the MOXO-dCPT and consequently enhancing its utility in clinical settings). Finally, we aimed to establish cutoffs for diagnosing ADHD based on the findings. These were meant to serve future validation studies that may, with time, pave the way for the integration of the MOXO-dCPT in daily clinical practice.

## 2. Materials and Methods

### 2.1. Participants

Adult ADHD patients and healthy controls participated in the study. They were undergraduate students who participated in the study for academic credit. An ADHD diagnosis (314.0x) based on the Diagnostic and Statistical Manual of Mental Disorders, Fifth Edition criteria (DSM-5; [41]) was mandated for all ADHD patients (clinical group). The participation of participants with ADHD in the study depended on submitting a documented diagnosis provided by a licensed clinician up to five years prior to study entry. The documented diagnoses were reaffirmed by a trained clinician upon study entry, using the Structured Clinical Interview for DSM-5 (SCID-5-RV; [42]). All participants had normal or corrected-to-normal vision. Participants in the ADHD group with past or present learning disabilities and/or neuropsychiatric disorders apart from ADHD were excluded from the study. Healthy controls were also excluded if reporting attentional impairment, operationalized as total score ≥ 37 in the adult ADHD self-report scale (ASRS). Abnormal eye movement values (i.e., duration of blinks) were noted in the data of three participants. Their data were therefore discarded. Overall, data of 43 ADHD patients and 42 healthy controls were included in the final analyses (*N* = 85), partially overlapping with those included in Berger, et al. [43] and Lev, Braw, Elbaum, Wagner and Rassovsky [28]. Ethical clearance for the study was obtained from the institutional ethics committee, and written consent was obtained from all participants.

### 2.2. Materials

#### 2.2.1. MOXO-dCPT

**Figure 1:**
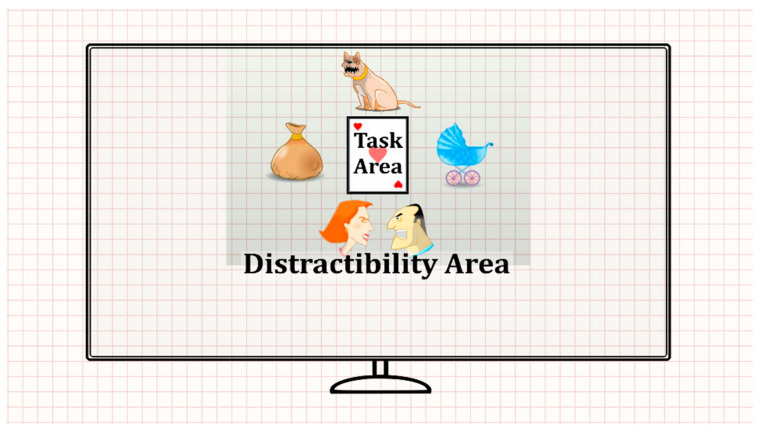
Participant field of view (FOV) while performing the MOXO-dCPT, divided into distractibility (gridded) and target areas of interest (AOIs).

The task consisted of eight blocks, each comprising 59 trials (total duration = 18.7 min). In each trial, a stimulus card (target/non-target) was presented in the screen center, followed by a blank screen (see Figure 1). Participants were requested to press the spacebar when a target appeared (a card with three red hearts) while refraining from responding when a non-target appeared. This was done while trying to ignore a series of visual and/or auditory distractors. The eight blocks of the MOXO-dCPT were divided into four stages, based on the presence and type of distractors: (a) No distractors: Blocks 1 and 8 were free of distractors. (b) Visual distractors: Blocks 2 and 3 included visual distractors (e.g., animation of a barking dog) that appeared above, below, or to the stimulus card’s sides. (c) Auditory distractors: Blocks 4 and 5 included auditory distractors related to the visual distractors described earlier (e.g., the sound of a barking dog). (d) Combined (visual and auditory) distractors: Blocks 6 and 7 had visual and auditory distractors that appeared simultaneously (e.g., animation of a barking dog paired with the sound of a bark).

#### 2.2.2. Eye Tracking Apparatus

An EyeLink 1000 eye tracker (SR Research Ltd., Mississauga, Ontario Canada) was selected for its high spatial and temporal accuracy [44] and due to its extensive use in scientific research [45]. Binocular eye movements were recorded at a 250-Hz sampling rate and an accuracy of approximately 0.5°, considered high compared to other eye trackers. See Table 3 in Gibaldi, Vanegas, Bex and Maiello [44] for a comparison of the Eyelink system to other commercially available eye trackers. Participants were calibrated using an EyeLink 13-point calibration procedure, and a chin rest stabilized their heads. The eye tracking apparatus also included a host PC and display PC. The first tracked and computed the participants’ gaze position, while the latter displayed the stimuli (i.e., MOXO-dCPT). The stimuli were presented on an Alienware OptX 23” display screen with 1920 × 1080 resolution. The eye-to-screen viewing distance was approximately 60 cm, and black curtains were placed behind the computer screen to minimize external distractions. We used Data Viewer-Eyelink Software (SR Research Ltd., Mississauga, ON, Canada) with its commonly used eye movement parsing algorithm. For further details, see a comparison of the data viewer’s parsing with R’s “Gazepath” package in van Renswoude, et al. [46].

### 2.3. Procedure

The participants underwent the SCID-5-RV and then filled out a demographic/health questionnaire, the adult ADHD self-report scale (ASRS), and the Wender Utah rating scale (WURS) short form. The ASRS [47,48] includes 18 items that cover adult ADHD symptoms, while the 25 items of the WURS retrospectively evaluate the presence and severity of childhood ADHD symptoms [49]. Participants were then moved to a sound-isolated room, were calibrated to the eye tracker, and performed the MOXO-dCPT while their eyes were being monitored.

### 2.4. Data Analysis

#### 2.4.1. Preliminary Procedures and Analysis

Four types of responses were recorded while the participants performed the MOXO-dCPT: (a) Attention: Correct responses during stimulus presentation or the inter-stimulus interval (ISI) following it. (b) Timeliness: Correct responses only during the time in which the target stimulus was present on the screen. (c) Impulsivity: Commission errors during the time in which a non-target stimulus was presented. (d) Hyperactivity: Commission errors that were not coded as impulsive. These included multiple keystrokes in response to a target stimulus, for example. Four indices were then created, expressing the participant’s percentage of responses in each of the response categories out of the maximum possible during the task.

The participants’ field of view (FOV) was divided into two areas (see Figure 1): (a) Task AOI: The AOI containing the stimulus card (i.e., target/non-target stimuli). (b) Distractibility AOI: All other FOV areas irrelevant to the task. The distractibility AOI included the visual distractors, white screen area, and area outside the screen (see the gridded area in Figure 1). The eye movement distractibility scale was calculated as the proportion of time spent gazing at the distractibility AOI, relative to all areas total gaze durations [50]. This eye movement distractibility scale operationalized the extent to which visual attention was directed toward areas irrelevant for success in the CPT.

The groups were compared in demographic and clinical variables (see Table 1) using independent-samples *t*-tests and chi-square analyses (for parametric and non-parametric variables, respectively). Welch’s unequal variance *t*-test was used when the assumption of group’s homogeneity of variance was not met [51]. All analyses were based on two-tailed hypotheses with *p* < 0.05 marking statistical significance. The study’s dataset is available from the authors upon request.

#### 2.4.2. Primary Analysis (Complete MOXO/All Stages)

ADHD patients and controls were compared in the eye movement distractibility scale using an independent-samples *t*-test and the conventional MOXO-dCPT indices using a multivariate analysis of variance (MANOVA) with a between-subjects factor of group (see Table 2). We calculated effect sizes according to the generally acceptable indices for each statistical analysis: Cohen’s *d* for the *t*-tests and partial eta-squared (η^2^_p_) for the ANOVAs (i.e., MANOVA and repeated-measures ANOVA) [52]. To enable the interpretation of effect sizes, despite being on different scales, we used Cohen’s effect size categorizations for group comparisons [53,54]. Cohen’s *d*: small effect = 0.2–0.5; moderate effect = 0.5–0.8; large effect > 0.8. Partial eta-squared (η^2^_p_): Small effect = 0.01; moderate effect = 0.06; large effect = 0.14.

**Figure 2:**
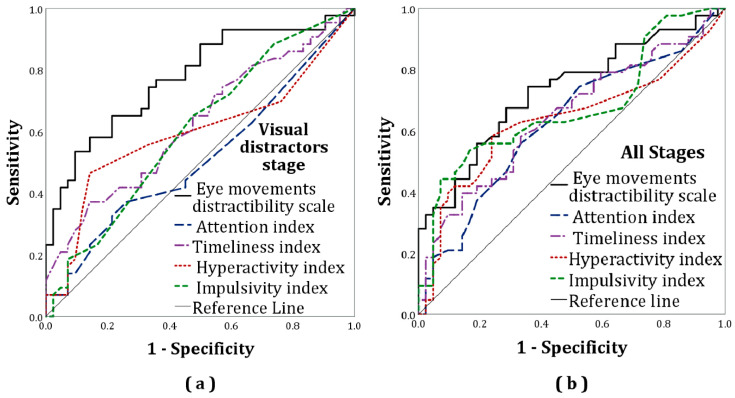
Receiver operating characteristic (ROC) curves for the eye movement distractibility scale and the MOXO-dCPT indices: (**a**) All stages; (**b**) visual distractors stage (eye movement distractibility scale: AUC = 0.78, attention: AUC = 0.51, timeliness index: AUC = 0.63, hyperactivity: AUC = 0.59, impulsivity: AUC = 0.60). Additional information regarding the visual distractors stage is presented later (see “Section 2.4.3. Exploratory Analysis”).

The classification accuracies of the eye movement distractibility scale and conventional MOXO-dCPT measures were assessed using a receiver operating characteristic (ROC) curve (see Figure 2). An ROC curve is a plot of true positive rates (sensitivity) against false positive rates (i.e., 1—specificity). The area under the curve (AUC) of these analyses was used to measure group discrimination capacity. Its range was 0.5–1.0, with larger values indicating better discrimination capacity. The AUC’s discrimination capacity was qualitatively interpreted following Hosmer, et al. [55] and indices of classification accuracy (i.e., sensitivity and specificity) for the eye movement distractibility scale were calculated for various cutoffs.

Next, a two-stage hierarchal logistic regression was performed, aiming to determine whether the eye movement distractibility scale could enhance the predictive power of the conventional MOXO-dCPT indices (see Table 3). The first regression model included the MOXO-dCPT indices, while the second model included both the indices and the eye movement distractibility scale. A default probability cutoff of 0.5 was used to classify participants based on the regression model’s predicted values. Multicollinearity was assessed before performing the regression analysis using a two-pronged approach. First, Pearson product-moment correlations between the MOXO-dCPT indices and the eye movement distractibility scale were performed. The correlation matrix was inspected with an *r* > 0.08 criterion used as a multicollinearity indicator. Second, multicollinearity was further assessed using the variance inflation factor criterion (i.e., VIF < 4; [56]). Note that the analyses of the visual distractors stage are presented in the exploratory analysis subsection.

**Figure 3:**
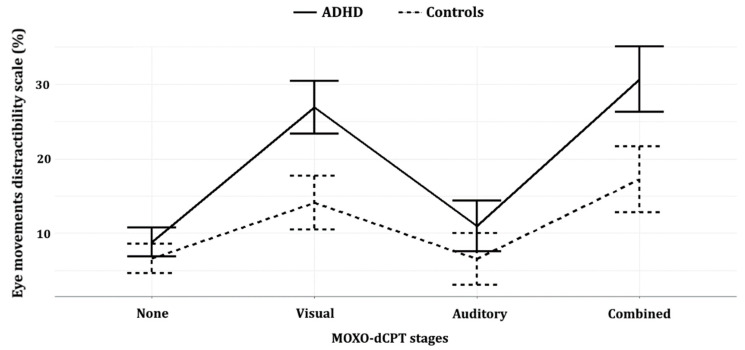
Eye movement distractibility line plot, of the four distractor-type stages. Error bars represent standard error (SE) of the repeated-measures ANOVA analysis (see Table 4).

Lastly, a repeated-measures two-way ANOVA was performed to test which stage (i.e., distractor type) in the MOXO-dCPT the eye movement distractibility scale best differentiated the groups (see Table 4). The analysis included *group* (ADHD/controls) as a between-subjects factor and *type of distractor* (non/visual/auditory/combined visual–auditory) as a within-subjects factor. Note in this regard that a repeated-measures two-way ANOVA was chosen because each participant performed the four stages of the MOXO-dCPT which differ in the type of distractor they employ (i.e., non/visual/auditory/combined visual–auditory). A line chart was produced (see Figure 3) to represent the results of the repeated-measures ANOVA. Error bars were added to the figure as the ANOVA’s standard errors (SE).

#### 2.4.3. Exploratory Analysis (Visual Distractors Stage Only)

Since the previous repeated-measures ANOVA analysis indicated that the visual distractors stage best differentiated the groups (see Table 4), we repeated all the performed analyses from the Section 2.4.2. Primary Analysis section, solely for the visual distractors stage. The visual distractors stage reanalysis included a *t*-test and a MANOVA (see Table 2), an ROC curve (see Figure 2), and a two-stage hierarchal logistic regression (see Table 3).

**Figure 4:**
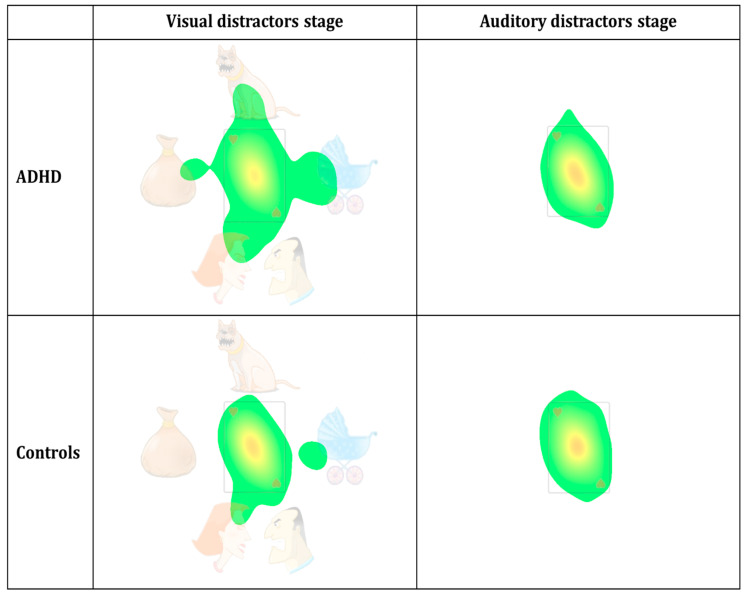
Heat maps of the eye distractibility scale, divided according to group (ADHD/controls) and MOXO-dCPT stages (visual/auditory).

**Figure 5:**
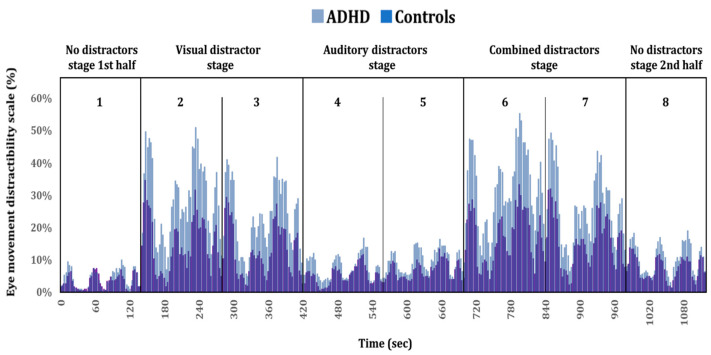
Timeline plot comparing eye movement distractibility scale of ADHD patients and controls. Four distractors stages (non, visual, auditory, and combined) are presented at the top section of the chart, subdivided into blocks one to eight. Each stage includes two consecutive blocks, except for the no distractors stage (blocks 1 and 8).

Density heat maps (see Figure 4), based on the groups’ aggregated fixations’ durations (also termed attention maps) were constructed, using SR-research Data-Viewer software (SR Research Ltd.). The red-yellow-green color scale represents visual attention (red indicates more attention), calculated as the proportion of summed durations of fixations towards the distractibility AOI, relative to the total fixations’ durations. As an initial reference point, we used the heat maps’ parameter setting by Ginsberg, et al. [57], which appeared to be compatible with our goals. For additional information, see Holmqvist and Andersson [58] and SR_Research [59]. Finally, to gain a better temporal resolution of the group difference in the eye movement distractibility scale, a timeline plot was produced (see Figure 5) by dividing the timeline into one second’s bins. For each bin (second), we calculated the percentage of all samples that fell outside the card area (i.e., distractibility AOI) across the total number of samples. This calculation was practically equivalent to the eye movement distractibility scale.

## 3. Results

The analyses were divided into two main sections. The first, under “Primary Analysis (Complete MOXO/All Stages)” (Section 3.2), presents the planned analyses for all four distractors stages as one entity (i.e., the complete MOXO-dCPT). The second section of analyses can be found under “Exploratory Analysis (Visual Distractors Stage Only)” (Section 2.4.3) and pertains to the results derived from the repeated-measures ANOVA (see Table 4), which suggested that analyzing only the visual distractors stage may lead to a better outcome. Thus, the “Exploratory Analysis” used the same analysis as in the “Primary Analysis”; however, there the analysis was applied only to the visual distractors stage.

### 3.1. Preliminary Analysis

As expected, ADHD patients had significantly higher total scores in the WURS 25-items, and ASRS (i.e., reported a more significant impairment) compared to healthy controls (*p* < 0.01), while no significant group differences were found in demographic variables. See Table 1.

### 3.2. Primary Analysis (Complete MOXO/All Stages)

There was a significant group difference with a large effect size in the eye movement distractibility scale; ADHD patients spent more time gazing at the distractibility AOI than controls (*p* < 0.01). There were also significant group differences in conventional MOXO-dCPT indices, *L* = 0.70, *F*(4, 80) = 4.81, *p* = 0.02. Post hoc analyses indicated that ADHD patients performed significantly worse than controls in the timeliness and impulsivity indices (*p* = 0.01 and *p* < 0.01; respectively). See Table 2.

ROC curve analyses indicated acceptable discriminative capacity of the eye movement distractibility scale (AUC = 0.73, *p* < 0.001), while that of the attention, timeliness, hyperactivity, and impulsivity indices were poor (AUC = 0.63, *p* = 0.04; AUC = 0.65, *p* = 0.01; AUC = 0.63, *p* = 0.03; AUC = 0.67, *p* = 0.003; respectively). See Figure 2 and Table 3.

All correlations between the predictors in the logistic regression were <0.08 and had VIF values <4, suggesting that multicollinearity likely did not impact the logistic regression’s findings. The first regression model significantly predicted group membership (*p* < 0.01), correctly classifying 69.8% ADHD patients and 71.4% controls. The stage 2 model, which added the eye movement distractibility scale to the predictors, significantly predicted group membership, *p* < 0.01. The model correctly classified 64.3% ADHD patients and 67.4% controls. Out of the predictors, only the eye movement distractibility scale and the impulsivity index significantly contributed to group prediction (*p* = 0.03, *p* = 0.01; respectively). Notably, the addition of the scale significantly contributed to group membership prediction beyond that of the conventional indices (i.e., first regression model), *p* = 0.02. See Table 3.

The repeated-measures ANOVA yielded significant *group* (ADHD/controls) and *type of distractor* (non/visual/auditory/combined) main effects, as well as a significant *group* X *type of distractor* interaction (*p*s < 0.01). Bonferroni multiple-comparison post hoc analyses yielded significant group differences during the visual and combined visual–auditory distractors stages (*p*s < 0.01; Cohen’s *d* = 1.11, Cohen’s *d* = 0.94; respectively), while the group differences in the no distractors and auditory distractors stages were not significant (*p* = 0.116 and *p* = 0.07; respectively). Notably, the effect size in the visual distractors stage (Cohen’s *d* = 1.11) was larger than that of the combined distractors stage (Cohen’s *d* = 0.94). See Table 4 and Figure 3.

The independent-samples *t*-test reanalysis for solely the visual distractors stage yielded similar results as found for the analysis of the full CPT, namely a significant group difference with an even higher effect size (Cohen’s *d* = 1.11). The MANOVA’s reanalysis showed no significant group differences in the MOXO-dCPT indices *F*(4,80) = 2.234, *p* = 0.07. The ROC reanalysis for the visual distractors stage indicated acceptable discriminative capacity of the eye movement distractibility scale (AUC = 0.78, *p* < 0.01). Regarding the indices, only the timeliness index showed significant, though poor, discriminative capacity (AUC = 0.63, *p* < 0.01). All other indices showed poor discriminative capacities (attention: AUC = 0.51, *p* = 0.83; hyperactivity: AUC = 0.59, *p* = 0.14; impulsivity: AUC = 0.60, *p* = 0.14). See Table 2.

Multicollinearity was not indicated by the preliminary analysis for the logistic regression that focused on the visual distractors stage. The first regression model (conventional indices as predictors) did not significantly predict group membership (*p* = 0.06), while correctly classifying 55.8% ADHD patients and 61.9% controls. The second regression model, which also included the eye movement distractibility scale, significantly predicted group membership, *p* < 0.01. The second model correctly classified 69.8% ADHD patients and 69.1% controls. Notably, the eye movement distractibility scale significantly contributed to group prediction over the first model, *p* < 0.01. Out of five predictors, only the eye movement distractibility scale’s coefficient contributed significantly to group prediction, *p* < 0.01. See Table 3. The interested reader can find cutoffs for the eye movement distractibility scale and conventional indices in the Supplementary Materials. See Table S1.

Aggregated density duration heat maps were generated for the visual distractors stage and auditory distractors stage using a Gaussian filter with a standard deviation of 0.6, a low activity cutoff of 3.4%, and a duration density maximum value of 30% (full red represents duration density of 30% and above). As evident in Figure 4, the red-yellow area at the center is smaller and with weaker colors, while the green area outside the card is larger, in the ADHD patient group compared to controls. Notably, these group differences appear solely in the visual distractors stage.

Figure 5 presents the group differences in visual attention using better temporal resolution. Taken together, Figure 4 and Figure 5 visually represent the group differences described earlier, indicating that ADHD patients tend to shift their attention away from the task area and towards the distractibility AOI compared to controls. See Figure 5.

## 4. Discussion

The current study assessed the utility of integrating an eye tracker with the MOXO-dCPT, a commercially available CPT. As only the center of the screen displayed the stimuli relevant for success in the task (i.e., target and non-target), gazing outside this AOI was counterproductive and could only impair performance. Therefore, the gaze direction measures were optimally suited to assess the distractibility and response inhibition deficits that are key characteristics of ADHD, in an intuitive and face-valid manner [60]. This clinical utility was evident in the current study. More specifically, the study’s primary eye movement measure, termed the eye movement distractibility scale, measured the proportion of time spent gazing outside the center of the screen. This measure of the allocation of attention to irrelevant stimuli (i.e., distractibility) significantly differentiated the groups. Its discriminative capacity was stronger than each of the MOXO-dCPT conventional indices (i.e., AUC in ROC curve analyses of 0.730 compared to 0.673 of the strongest of the conventional indices, impulsivity). Furthermore, the scale significantly enhanced the MOXO-dCPT’s discriminative capacity, compared to the sole use of conventional indices, corresponding to the findings of Fried, Tsitsiashvili, Bonneh, Sterkin, Wygnanski-Jaffe, Epstein and Polat [27] which utilized the TOVA CPT. From a cognitive standpoint, the findings can indicate both increased distractibility and response disinhibition among ADHD patients. Regarding the latter, novel studies indicate the utility of eye movement analyses to detect even subtle impairment in response inhibition. For example, gaze direction measures efficiently differentiated ADHD patients from controls in the Stroop test, a response inhibition task [61]. Similarly, ADHD patients had more difficulty than healthy controls in suppressing saccades toward the stimulus in the anti-saccade task [22,24], a task in which participants were instructed to make a saccade, a rapid eye movement to a specific location, away from a suddenly appearing stimulus [62]. Overall, the findings correspond to the well-established failure of ADHD patients to ignore distractors in daily life [63,64], strengthening the ecological validity of the MOXO-dCPT.

This sensitivity of the eye movement measures to detect subtle response inhibition deficits was further capitalized upon in the current study by assessing the differential utility of the MOXO-dCPT’s distractors. The MOXO-dCPT is unique in utilizing different types of “real-world” dynamic distractors to magnify cognitive impairment that may otherwise evade detection. The analyses of the MOXO-dCPT stages indicated that visual distractors (i.e., blocks 2 and 3) had the strongest discriminative capacity. During this stage, the groups significantly differed in the eye movement distractibility scale and it had stronger discriminative capacity than each of the conventional indices (AUC = 0.775). This was mirrored in the scale’s cutoffs, which could be set to enhance either sensitivity or specificity. The former was useful when screening for ADHD, while the latter had utility when false positives should be minimized (e.g., more thorough evaluations). For example, 88.4% of the ADHD patients spent 10.8% or more of their time gazing at the distractibility AOI, making the cutoff useful for screening purposes. On the other extreme, spending 39.4% or more gazing at the distractibility AOI was associated with perfect specificity (i.e., no false positives), though sensitivity was poor (23.3%). The interested reader can find cutoffs that either prioritize specificity (i.e., reduce false positives) or sensitivity (i.e., useful in screening for ADHD) for the eye movement distractibility scale in the Supplementary Materials. See Table S1. Importantly, the eye measures enhanced group prediction compared to the sole use of the conventional MOXO-dCPT indices. While integrating the conventional indices during the visual distractors stage correctly classified 55.8% ADHD patients and 61.9% controls, integration of the indices with the eye movement distractibility scale classified an additional 14% of the ADHD patients (i.e., sensitivity = 69.8%), while also raising the number of correctly diagnosed healthy controls (i.e., specificity = 69.1%).

The discrimination capacity of the visual distractors stage was closely followed by that of the combined distractors stage, suggesting that visual distractors drive these group differences. This mirrors earlier findings regarding the differential utility of distractors in the MOXO-dCPT [38,39]. For example, Berger and Cassuto [38] concluded that “ADHD adolescents were more distracted by pure visual distractors and by the combination of distractors than by pure auditory ones” (see abstract). On a more general level, the current study’s findings have practical and theoretical implications. Regarding the former, we believe that the MOXO-dCPT eye tracker integrated system, and similar systems, may prove useful in both clarifying the cognitive profile of the examinees and in aiding diagnostic decisions. As noted earlier, integration of the conventional MOXO-dCPT indices with the eye movement distractibility scale enhanced the MOXO-dCPT discrimination capacity compared to the sole use of conventional indices, placing it at the moderate-high range (see Table 1 in [13]) and suggesting its clinical utility. At the same time, it should be noted that the scale did not reach the 80% sensitivity/specificity biomarker criterion suggested by the World Federation of Societies of Biological Psychiatry, WFSBP [19]. In addition, the scale’s strength compared to other potential biomarkers of ADHD has not been established (e.g., EEG theta/beta ratio [EEG-TBR]; [65]), including those based on eye movement analysis. For example, analysis of eye vergence responses while adult participants performed an attentional task appeared to provide a somewhat stronger discriminative capacity than that found in the current study (sensitivity = 79% and specificity = 75%; [66]). This emphasizes the need for more research, both to validate the current findings and to compare the utility of eye movement measures, either alone or when combined with conventional CPT measures, to other biomarkers of ADHD. While planning future research avenues, it should be kept in mind that the use of a commercially available task, as used in the current study, offers to ease any future integration of such measures in daily clinical practice. It also enhances the ability to compare the findings of such studies, in contrast to those which use tasks constructed specifically for each study. These factors may offset minor advantages in discriminative capacity that are suggested by other, sometimes invasive, biomarkers. An additional practical implication of the findings stems from the fact that the visual distractors stage included the second and third blocks of the MOXO-dCPT. This tentatively implies that the task may be shortened with relative ease and a minimal decrease in efficacy. More specifically, we may reduce 62.5% of the MOXO-dCPT’s length (i.e., shortening it from 18.7 min to approximately 7 min) by removing the last five blocks. This would leave only the first block, which included no distractors, and blocks 2 and 3 (i.e., the visual distractors stage). This would allow participants to be acquainted with the CPT and practice it during the first block before performing the main part of the task (i.e., the visual distractors stage). As is evident from the current study’s findings, this would lower somewhat the efficacy of conventional indices. However, this would be compensated by the enhanced discriminative capacity of the eye movement distractibility scale, as well as that stemming from its integration with the MOXO-dCPT’s conventional indices. These promise adequate discriminative capacity, although additional research is needed to validate these findings. If supportive, such research may pave the way to shortening the MOXO-dCPT with practical benefits which include streamlining clinical practice by reducing time expenditure and costs (e.g., technicians’ costs for administering the CPT). From a theoretical perspective, studies have begun to assess factors influencing CPT performance. More specifically, there are initial indications that the use of visual stimuli in CPTs leads to faster response times and more false positives than when auditory stimuli are used [67,68]. Response inhibition may therefore be modality-specific [40], possibly underlying the developmental delay evident in ADHD and the asynchronous maturation of hearing and vision [69]. As the growing literature suggests, behavioral and neural processes involved in response inhibition depend on the modality of the stimuli used in the CPTs. The current study indicates that the distinction between auditory and visual stimuli may also impact the effects of distractors in CPTs, and not only the target/non-target stimuli used in the CPT. It therefore addresses the growing literature that aims to deconstruct the factors that affect response disinhibition of ADHD patients and the factors that enhance the discriminative capacity of the CPTs. These may lead to a theoretically driven construction of future CPTs.

The current study’s strengths include the use of a face-valid gaze direction measure which is particularly appropriate for exploring the inattention and response inhibition deficits of ADHD patients. The MOXO-dCPT’s ecological distractors in this regard are particularly attractive for such an eye tracker-CPT integration. Limitations of the current study include: (a) The MOXO-dCPT does not randomize the stages, possibly confounding the distractor type and order of stages. This, for example, may have led to the enhanced utility of the visual distractors stage as compared to the combined distractors stage (i.e., blocks 2–3 vs. blocks 6–7). Future studies may consider collaborating with the MOXO-dCPT publishers to create a version of the CPT in which the stages are randomized. Thus, this version will differ from that used in clinical practice, which should be acknowledged. (b) The sample size of the current study did not allow a comparison between ADHD subtypes (inattentive, hyperactive/impulsive, and combined). This is unfortunate, as these subtypes may differ in their cognitive profile [60]. Comparing ADHD subtypes in future research would be of interest, especially considering that adults with ADHD have prominent inattentive symptoms [70]. (c) Generalizing the findings to other ADHD subpopulations should be done cautiously, as the ADHD patients were undergraduate students and therefore represent highly functioning patients [71]. Furthermore, as ADHD participants were required to have a prior diagnosis, students with ADHD who were not previously diagnosed were not represented in the study. Participants with substantial comorbid psychopathology were excluded from the study, an additional constraint on the generalizability of the findings, considering the high prevalence of comorbidity among ADHD patients [72]. This limitation can be addressed in future studies by assessing examinees undergoing assessments in clinical settings, as well as assessing non-academic ADHD patients and those with comorbid psychopathology. Additional lines of research can easily be developed in future research. For example, it would be of interest to assess the association between eye movement measures in CPT and attentional networks which have been previously suggested (e.g., alerting, orienting and executive control; [73]), as well as in vivo brain activity. Especially relevant for further research is the distinction between endogenous and exogenous spatial attentional systems, and it would be of interest to explore whether, for example, the profile of ADHD patients reflects increased susceptibility of external events to attract spatial attention via bottom-up or exogenous mechanisms [74]. Finally, comparing ADHD patients with patients with other neuropsychiatric disorders is in order.

## 5. Conclusions

The current study assessed the utility of an eye tracker-integrated CPT, while analyzing each stage of the MOXO-dCPT (i.e., types of distractors). ADHD patients were found to spend more time gazing outside the task area compared to healthy controls, and this was particularly notable when visual distractors were utilized. The MOXO-dCPT’s ecological distractors therefore impact the performance of ADHD patients in the task and they have difficulty in suppressing spontaneous eye movements towards such distractors. These findings have both practical and theoretical implications. They suggest that the discriminative capacity of the MOXO-dCPT, and perhaps other CPTs, may be enhanced and clinical decision-making improved by integrating data derived from conventional indices and eye movement measures. They also indicate that the MOXO-dCPT may be shortened with implications regarding the cost and time of evaluating those with ADHD or suspected to have the disorder. From a theoretical perspective, the enhanced utility of the visual distractors adds to the accumulating literature on distinctions between visual and auditory stimuli in CPTs, possibly enhancing our ability to theoretically drive the development of CPTs. The overall findings nicely align with the rapidly expanding research of biomarkers in psychiatry and the growing efforts to enhance diagnostic precision in the field [18,75]. CPTs, neuropsychological tasks which are widely used in clinical settings, are ideal in this sense as a laboratory for the development of the validation of these novel endeavors. The declining costs, increased ease of usability, and reduced bulkiness of eye tracking equipment in recent years constitute a further impetus for their integration with future CPT research. This may pave the way for their incorporation in daily clinical practice with time.

## Figures and Tables

**Table 1:** Demographic and clinical data of attention-deficit/hyperactivity disorder (ADHD) patients (*n* = 43) and healthy controls (*n* = 42).

Measures	ADHD Patients	Healthy Controls	Statistical Analyses	Effect Size
	Mean ± SD	Mean ± SD	Parametric	Cohen’s *d*
Age (years)	23.84 ± 2.28	23.88 ± 2.58	*t*(83) = 0.08, *p* = 0.93	0.02
Education level (years)	13.09 ± 1.15	12.83 ± 2.38	*t*(83) = 0.64, *p* = 0.52	0.14
WURS 25-items (no.)	30.51 ± 13.83	17.90 ± 10.10	*t*(83) = 4.79, *p* < 0.01	1.04
ASRS (no.)	31.95 ± 11.13	21.36 ± 6.47	*t*(83) = 5.35, *p* < 0.01	1.16
	Frequencies	Frequencies	Non-parametric	OR
Gender (male/female)	17/26	11/31	χ^2^(1) = 1.71, *p* = 0.19	1.84
Dominance (right/left)	39/4	35/6	χ^2^(1) = 0.57, *p* = 0.45	1.67
Glasses (yes/no)	8/35	5/37	χ^2^(1) = 0.74, *p* = 0.39	1.69

Note: ASRS: Adult ADHD self-report scale; WURS: Wender Utah rating scale; OR: Odds ratio.

**Table 2:** Analyses of group differences in the eye movement distractibility scale and conventional MOXO-dCPT indices, presented for the all MOXO-dCPT stages and the visual distractors stage.

Measures		ADHD (Mean ± SD)	Controls (Mean ± SD)	Statistical Analyses	Effect Size
All stages	Eyes movements scale (%)	19.28 ± 11.03	11.06 ± 6.89	*t*(83) = 4.11, *p* < 0.01	*d* = 0.89
	Attention (%)	95.52 ± 5.71	97.33 ± 4.16	*F*(1, 83) = 2.78, *p* = 0.10	η_p_^2^ = 0.03
	Timelines (%)	73.56 ± 14.57	80.48 ± 10.62	*F*(1, 83) = 6.23, *p* = 0.01	η_p_^2^ = 0.08
	Hyperactivity (%)	3.76 ± 4.16	2.14 ± 4.05	*F*(1,83) = 4.96, *p* = 0.29	η_p_^2^ = 0.06
	Impulsivity (%)	5.28 ± 3.38	3.14 ± 2.2	*F*(1,83) = 12.5, *p* < 0.01	η_p_^2^ = 0.13
Visual distractors stage	Eyes movements scale (%)	26.94 ± 13.63	14.11 ± 9.06	*t*(83) = 5.10, *p* < 0.01	*d* = 1.11
	Attention (%)	94.94 ± 8.26	96.43 ± 5.40	*F*(1, 83) = 0.96, *p* = 0.33	η_p_^2^ = 0.01
	Timelines (%)	67.85 ± 16.11	74.61 ± 12.11	*F*(1, 83) = 4.77, *p* = 0.03	η_p_^2^ = 0.05
	Hyperactivity (%)	4.41 ± 5.35	2.63 ± 3.64	*F*(1, 83) = 3.22, *p* = 0.08	η_p_^2^ = 0.04
	Impulsivity (%)	4.82 ± 3.49	3.61 ± 3.33	*F*(1, 83) = 2.7, *p* = 0.10	η_p_^2^ = 0.03

**Table 3:** Two-stage hierarchical logistic regressions (*N* = 85), for all MOXO-dCPT stages and the visual distractors stage. The first model includes the four conventional indices and the second model adds the eye movement distractibility scale.

	Model Statistics		Coefficients Statistics				
	Model *χ*^2^(df), *p*	2nd Stage (Δ) *χ*^2^(df), *p*	*β*	ES	OR	OR CI	Wald
**Logistic Regression I: All MOXO-dCPT Stages**							
Stage 1	18.73(4), *p* < 0.01	____					
1. Attention			0.075	0.08	1.08	0.93–1.23	0.93, *p* = 0.34
2. Timeliness			−0.068	0.03	0.93	0.88–0.99	5.49, *p* = 0.02
3. Hyperactivity			−0.028	0.07	0.97	0.84–1.12	0.15, *p* = 0.70
4. Impulsivity			−0.338	0.12	1.40	1.12–1.76	8.47, *p* = 0.03
+ Constant			−3.252	6.33	0.04	____	0.26, *p* = 0.61
Stage 2	24.50(5), *p* < 0.01	5.77(1), *p* = 0.02					
1. Attention			0.060	0.08	1.06	0.91–1.25	0.56, *p* = 0.45
2. Timeliness			−0.046	0.03	0.96	0.90–1.01	2.27, *p* = 0.13
3. Hyperactivity			−0.072	0.08	0.93	0.80–1.09	0.80, *p* = 0.37
4. Impulsivity			0.295	0.12	1.34	1.07–1.69	6.42, *p* = 0.01
5. Eye movements scale			0.075	0.03	1.08	1.01–1.15	4.82, *p* = 0.03
+ Constant			−4.359	6.61	0.01	____	0.43, *p* = 0.51
**Logistic Regression II: Visual Distractors Stage**							
Stage 1	8.96(4), *p* = 0.06	^___^					
1. Attention			0.040	0.04	1.04	0.95–1.14	0.82, *p* = 0.34
2. Timeliness			−0.043	0.02	0.96	0.92–1.00	4.00, *p =* 0.05
3. Hyperactivity			−0.061	0.06	1.06	0.95–1.19	1.06, *p* = 0.30
4. Impulsivity			−0.085	0.08	1.09	0.94–1.26	1.24, *p* = 0.27
+ Constant			−1.325	3.57	0.27	____	0.13, *p* = 0.71
Stage 2	23.98(5), *p* < 0.01	15.02(1), *p* < 001					
1. Attention			0.051	0.05	1.05	0.96–1.16	1.08, *p* = 0.30
2. Timeliness			−0.011	0.02	0.99	0.94–1.04	0.20, *p* = 0.66
3. Hyperactivity			−0.014	0.06	0.99	0.87–1.12	0.05, *p* = 0.83
4. Impulsivity			0.049	0.08	1.05	0.89–1.24	0.35, *p* = 0.55
5. Eye movements scale			0.098	0.03	1.10	1.04–1.17	11.82, *p* < 0.01
+ Constant			**−6.252**	4.31	0.00	____	**2.10, *p* = 0.15**

Note. CI: Confidence interval (95%); OR: Odds ratio; SE: Standard error.

**Table 4:** Repeated-measures ANOVA for the eye movement distractibility scale: (**a**) Primary analysis; (**b**) post hoc analysis of the interaction effect using multiple pairwise comparisons with a Bonferroni correction.

**(a) Repeated-Measures ANOVA**						
**Factors**	***df* (83)**	***F***	***p***	**η_p_²**	**Pairwise Comparisons (Bonferroni)**	
*Group*	1	16.67	*p* < 0.01	0.17	Controls < ADHD	
*Distractors stage*	3	107.55	*p* < 0.01	0.56	Non < visual; Non = auditory; Non < combined; Auditory < visual; Auditory < combined; Visual < combined	
*Group* x *Distractors stages*	3	13.14	*p* < 0.01	0.14	Comparisons are detailed below	
**(b) Pairwise Comparisons (Bonferroni) for the *Group* X *Distractors Stage* Interaction**						
*Distractors stage*	ADHD (*n* = 43)		Controls (*n* = 42)		*p*	Cohen’s *d*
	Mean	SD	Mean	SD		
Non	8.81	6.82	6.63	5.8	*p* = 0.12	0.34
Visual	26.94	13.63	14.11	9.06	*p* < 0.01	1.11
Auditory	11.00	12.38	6.56	9.72	*p* = 0.07	0.40
Combined	30.68	16.11	17.26	12.24	*p* < 0.01	0.94

Note. ANOVA: Analysis of variance; df: Degrees of freedom; SD: Standard deviation; Combined: Auditory + visual distractors. Section 2.4.3. Exploratory Analysis (Visual Distractors Stage Only).

## Supplementary Materials

The following are available online at https://www.mdpi.com/1424-8220/20/21/6386/s1, Table S1. Visual distractors stage: Classification accuracy of outcome measures.
