# Supplementary material for: Attention-Deficit/Hyperactivity Disorder (ADHD): Integrating the MOXO-dCPT with an Eye Tracker Enhances Diagnostic Precision
Source: Sensors (Basel). 2020 Nov 9;20(21):6386. doi: 10.3390/s20216386 (PMC7664925; doi:10.3390/s20216386)
Supplement: Supplementary file 1 [file sensors-20-06386-s001.pdf]

Supplementary Materials

Table S1. Visual distractors stage: Classification accuracy of outcome measures.

Eye Distractibility Scale			Attention Index			Timeliness Index			Hyperactivity Index			Impulsivity Index		
**Cutoff (%)**	**SPEC (%)**	**SENS (%)**	**Cutoff (%)**	**SPEC (%)**	**SENS (%)**	**Cutoff (%)**	**SPEC (%)**	**SENS (%)**	**Cutoff (%)**	**SPEC (%)**	**SENS (%)**	**Cutoff (%)**	**SPEC (%)**	**SENS (%)**
27.8	100	27.9	99.8	97.6	0	98.0	95.2	0	20.0	97.6	0	10.5	100	9.3
25.6	97.6	30.2	99.4	85.7	14	97.1	95.2	2.3	16.0	97.6	2.3	9.9	97.6	9.3
23.4	95.2	32.6	99.1	64.3	20.9	95.8	92.9	4.7	14.0	97.6	4.7	9.0	95.2	9.3
22.6	92.9	34.9	98.7	52.4	25.6	94.5	92.9	9.3	11.8	95.2	4.7	8.3	95.2	16.3
22.4	88.1	34.9	98.3	45.2	34.9	93.6	90.5	9.3	11.2	95.2	7.0	7.9	95.2	25.6
21.2	88.1	39.5	—	—	—	92.5	88.1	9.3	10.5	95.2	9.3	7.5	95.2	27.9
20.7	85.7	44.2	—	—	—	91.4	88.1	11.6	9.2	95.2	11.6	7.2	95.2	32.6
19.9	83.3	46.5	—	—	—	90.4	85.7	11.6	8.3	95.2	14.0	6.8	92.9	39.5
18.0	81.0	48.8	—	—	—	89.5	83.3	11.6	7.9	95.2	16.3	6.4	92.9	41.9
15.8	81.0	53.5	—	—	—	89.2	81	11.6	7.0	92.9	18.6	6.1	92.9	44.2
15.1	78.6	55.8	—	—	—	88.6	78.6	11.6	6.1	92.9	20.9	5.7	88.1	44.2
14.7	73.8	58.1	—	—	—	88.1	76.2	14	5.7	92.9	23.3	5.3	88.1	46.5
14.2	73.8	62.8	—	—	—	87.7	76.2	16.3	5.3	92.9	25.6	5.0	85.7	48.8
13.9	71.4	65.1	—	—	—	87.3	73.8	18.6	5.0	92.9	27.9	4.6	83.3	53.5
13.7	69.0	67.4	—	—	—	86.9	71.4	18.6	4.6	92.9	32.6	4.2	78.6	55.8
13.3	64.3	67.4	—	—	—	86.6	69.0	18.6	4.2	92.9	34.9	3.9	69	55.8
12.8	64.3	74.4	—	—	—	86.2	66.7	20.9	3.9	90.5	37.2	3.5	69	58.1
12.3	57.1	74.4	—	—	—	85.8	61.9	20.9	3.5	90.5	39.5	3.1	61.9	62.8
11.0	54.8	76.7	—	—	—	85.5	59.5	20.9	3.1	88.1	41.9	2.8	52.4	62.8
10.0	52.4	79.1	—	—	—	84.7	59.5	23.3	2.8	83.3	41.9	2.4	33.3	67.4
—	—	—	—	—	—	84.0	57.1	23.3	2.4	81	44.2	—	—	—
—	—	—	—	—	—	83.5	57.1	27.9	2.0	76.2	51.2	—	—	—
—	—	—	—	—	—	82.9	52.4	27.9	1.7	76.2	58.1	—	—	—
—	—	—	—	—	—	82.4	50	30.2	1.3	66.7	62.8	—	—	—
—	—	—	—	—	—	81.8	50	32.6	0.9	45.2	67.4	—	—	—

Note. SENS: Sensitivity; SPEC: Specificity.
